# Effect of Conduction Band Non-Parabolicity on the Nonlinear Optical Properties in GaAs/*Ga*_1−*x*_*Al_x_As* Double Semi-V-shaped Quantum Wells

**DOI:** 10.3390/ma12010078

**Published:** 2018-12-26

**Authors:** Zhi-Hai Zhang, Jian-Hui Yuan, Kang-Xian Guo, Elmustapha Feddi

**Affiliations:** 1 College of Physics and Electronic Engineering, Yancheng Teachers University, Yancheng 224007, China; 2 Department of Physics, Guangxi Medical University, Nanning 530021, China; 3 Department of Physics, College of Physics and Electronic Engineering, Guangzhou University, Guangzhou 510006, China; kxguo@gzhu.edu.cn; 4 ENSET de Rabat, Universite Mohamed V Souissi, B.P. 6207, Rabat-Institut, Rabat 10000, Morocco; e.feddi@um5s.net.ma

**Keywords:** non-parabolicity effect, optical properties, finite difference method, double quantum wells

## Abstract

In this paper, we investigate the effect of conduction band non-parabolicity (NPBE) on the third harmonic generation(THG), the linear and nonlinear intersub-band optical absorption coefficients (OACs) related with electronic states of double semi-V-shaped GaAs/*Ga*_1−*x*_*Al_x_As* quantum wells(QWs) by using the compact-density-matrix approach. Simultaneously, the work is performed in the position dependent effective mass in order to compute the electronic structure for the system by the finite difference and self-consistent techniques. We also compare the results with and without considering NPBE. It is found that: (1) the NPBE has a significant influence on the sub-band energy levels of double semi-V-shaped QWs, and (2) the amplitude and position of the resonant peaks of the THG and nonlinear OACs in the case of considering NPBE show complicated behavior due to the energy dependent effective mass m*(E) where the energy value was chosen self-consistently.

## 1. Introduction

Nowadays, the fundamental properties of low-dimensional semiconductor structures play an extremely important role in developing new technologies in various areas. Because of the quantum confinement of the charge carriers in low-dimensional semiconductor structures, these structures have remarkably light emitting efficiency compared to that in bulk materials. Among the important properties of the low-dimensional semiconductor structures, the nonlinear optical features are of great importance, since their applications can considerably improve the characteristics of different optoelectronics devices. During the last few years, many publications have appeared on different aspects of the nonlinear optical properties of low-dimensional semiconductor structures [1,2,3,4]. Simultaneously, with the technological progresses in the fabrication of semiconductor structures like Molecular Beam Epitaxy and Metal Organic Chemical Vapor Deposition [5,6,7,8], it has been made possible to fabricate variety of nanosized structures, such as quantum dot, quantum well (QW) and quantum wire. In particular, the semiconductor QW owing to their unique larger-band-gap are attractive both for fundamental research and applications in optoelectronics devices [9,10,11,12].

It is worth mentioning that the coupled double QW systems have many novel optical properties, which have been widely studied theoretically by several authors. For instance, Chen et al. studied the applied electric field on the nonlinear optical properties in symmetric and asymmetric double triangular QWs. The results show that the structure parameters of double QWs and applied electric field have a significant effect on the nonlinear optical properties, and the magnitude of the second harmonic generation is 1–2 orders of magnitude higher than that in step single QW system [13,14]. M.J. Karimi et al. studied the linear and nonlinear intersub-band optical absorption coefficients (OACs) and refractive index changes in asymmetric and symmetric double semi-parabolic QWs. The results show that the different values of the intersub-band energy interval and matrix elements have led to different physical behaviors for asymmetric and symmetric double semi-parabolic QWs. But, the total OACs of asymmetric double semi-parabolic QWs is usually greater than that of symmetric double semi-parabolic QWs [15,16]. L.E.G. Armas studied the Al concentration on the electric properties of coupled and uncoupled Al*_x_*Ga_1−*x*_As/AlAs/Al*_y_*Ga_1−*y*_As double QWs [17]. N. Angayarkanni et al. have studied the effect of laser field on the interband OACs in a strained GaAs/GaAlAs double QW system. The results show that the interband emission energy and the interband OACs depend strongly on the structure parameters of double QWs [18]. A. Keshavarz and N. Zamani have obtained the optimization of OACs in asymmetric double rectangular QWs by the use of the particle swarm optimization algorithm [19]. Z. Parang et al. have studied the nonlinear intersub-band optical absorption in double modified Pöschl-Teller QWs [20]. F. Ungan et al. have studied the effects of electric and magnetic fields on the OACs and refractive index changes in double inverse parabolic QWs and GaInNAs/GaAs double QWs [21]. T. Wecker et al. have studied the structural and optical properties in asymmetric cubic GaN/Al*_x_*Ga_1−*x*_N double QWs [22].

Among various confining potential profiles, the double semi-V-shaped potential has attracted some research attention because it has the tunable parameters of asymmetry degree. The nonlinear optical properties can be modified and controlled by tuning the structure parameters. In 2012, U. Yesilgul investigated the linear and nonlinear intersub-band OACs and refractive index changes in symmetric double semi-V-shaped QWs. The numerical results show that the geometrical parameters have a great effect on the optical characteristics [23]. In 2015, U. Yesilgul et al. calculated the effect of geometrical parameters on the linear, nonlinear OACs and refractive index changes of asymmetric double semi-V-shaped QWs [24]. However, by applying the proposed double semi-V-shaped potential we could not efficiently perform our calculations. In fact, the effect of conduction band parabolicity (PBE) is widely used in the calculation of the electron states close to the band edge of the conduction band based on the single-band effective mass. When the electron energy becomes much higher than the conduction band edge in the QW structure, the effect of conduction band non-parabolicity (NPBE) should be properly included. In this manuscript, we are giving a more suitable double semi-V-shaped potential, and the NPBE has been considered as well for more precise calculations.

**Figure 1:**
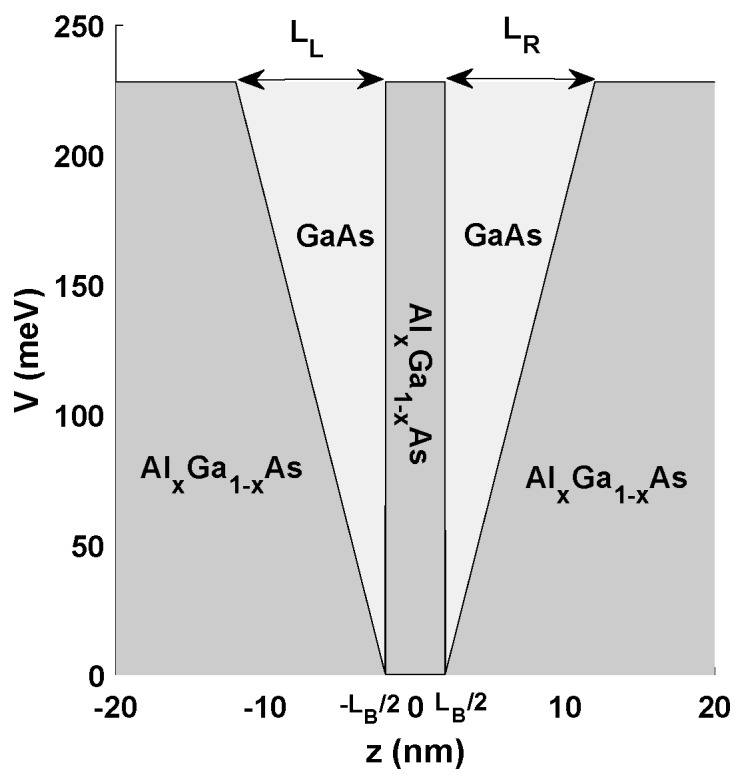
Geometric scheme of the GaAs/Ga_1−*x*_Al*_x_*As double semi-V-shaped quantum wells.

In the present work, a schematic diagram for modified double semi-V-shaped QWs system is given in Figure 1. We focus on the NPBE on the nonlinear OACs and THG in double semi-V-shaped QWs. The paper is organized as follows: In Section 2, we describe our theoretical model, and the eigenfunctions and eigenenergies of electron states are obtained using finite difference method. Our numerical results are discussed in Section 3, and a brief conclusions is presented in Section 4.

## 2. Theory

Let us consider an electron confined in the GaAs/*Ga*_1−*x*_*Al_x_As* double semi-V-shaped QWs. Within the non-parabolicity correction, the Schrödinger equation of this system can be written as

(1) $$\left[-\frac{\hbar^{2}}{2}\frac{\partial }{\partial z}\frac{1}{m^{*}\left(z,E_{n}\right)}\frac{\partial }{\partial z}+V\left(z\right)\right]\Psi_{n}\left(z\right)=E_{n}\Psi_{n}\left(z\right),$$

where V(z) is the double semi-V-shaped confining potential. $m^{*}\left(z,E_{n}\right)$ is non-parabolicity effective mass along the growth direction *z*, which is defined as [25,26,27]

(2) $$m^{*}\left(z,E_{n}\right)=m^{*}\left(z\right)\left(1+\frac{4\gamma m^{*}\left(z\right)E_{n}}{\hbar^{2}}\right),$$

where $\gamma =\frac{\hbar^{2}}{2m^{*}}{\left(1-\frac{m^{*}}{m_{0}}\right)}^{2}\frac{3+4\vartheta +2\vartheta^{2}}{3+5\vartheta +2\vartheta^{2}}\frac{1}{E_{g}}$, $\vartheta =\frac{\Delta }{E_{g}}$ (we take the zero of energy at the bottom of the conduction band), $E_{g}=1519$ meV is the energy gap, Δ=341 meV is the spin-orbit splitting, $m^{*}\left(z\right)$ is the band edge effective mass, which may vary with position due to the changes in materials composition over the length of the device.In this manuscript, we consider the non-parabolicity conduction band in $Ga_{1-x}Al_{x}As$ with aluminum concentration x=0.3, because this is the most typical alloy material [28,29], and in experimental data, the effective mass for $Ga_{1-x}Al_{x}As$ can be taken as $m^{*}\left(z\right)=\left(0.0665+0.0835x\right)m_{0}$ for 0≤x≤0.45, while for GaAs is $m^{*}\left(z\right)=0.067m_{0}$ ($m_{0}$ is the free electron mass) [30,31].

The double semi-V-shaped confining potential V(z) can be defined as

(3) $$\begin{array}{c} V\left(z\right)=\left\{\begin{array}{cc} \left(\frac{-z-L_{B}/2}{L_{L}}\right)V_{0} & -\frac{L_{B}}{2}-L_{L}<z<-\frac{L_{B}}{2}\\ V_{0} & -\frac{L_{B}}{2}\leq z\leq \frac{L_{B}}{2},z\geq \frac{L_{B}}{2}+L_{R},z\leq -\frac{L_{B}}{2}-L_{L}\\ \left(\frac{z-L_{B}/2}{L_{R}}\right)V_{0} & \frac{L_{B}}{2}<z<\frac{L_{B}}{2}+L_{R} \end{array}\right. \end{array}$$

here $V_{0}=0.6\Delta E_{g}^{\Gamma }\left(x\right)$ is the barrier height between GaAs and $Ga_{1-x}Al_{x}As$. $\Delta E_{g}^{\Gamma }\left(x\right)=1.155x+0.37x^{2}$ is the difference in the band gap energy of GaAs and $Ga_{1-x}Al_{x}As$ at the Γ point. $L_{L}$, $L_{R}$ and $L_{B}$ are the sizes of left-hand QW, the right-hand QW, and the central barrier, respectively.

The Schrödinger Equation (1) can be solved by using the finite difference method and iterative shooting techniques with adjusting the effective mass for each energy *E* [32,33], which may be discretized using the finite difference approximation to obtain

(4) $$-\frac{\hbar^{2}}{2\delta z^{2}}\left\{\frac{\Psi_{i+1}-\Psi_{i}}{m_{i+1/2}^{*}\left(z_{i+1/2},E_{n}\right)}-\frac{\Psi_{i}-\Psi_{i-1}}{m_{i-1/2}^{*}\left(z_{i-1/2},E_{n}\right)}\right\}+V_{i}\Psi_{i}=E_{n}\Psi_{i}.$$

where (i±1/2) are treated as being the mean of the two adjacent points.

After the electron energy levels *E* and their corresponding wave function $\Psi_{i}\left(z\right)$ in double semi-V-shaped QWs are obtained. Next, the nonlinear OACs and THG coefficients can be obtained by the compact-density-matrix method and the iterative procedure [34,35,36]. First, we consider that the system is excited by electromagnetic field $\vec{E}\left(t\right)=\tilde{E}e^{i\omega t}+\tilde{E}e^{-i\omega t}$. We obtain the time-dependent Liouville equation

(5) $$\frac{\partial \rho_{ij}}{\partial t}=\frac{1}{i\hbar }{\left[{\hat{H}}_{0}-\hat{M}\vec{E}\left(t\right),\hat{\rho }\right]}_{ij}-\Gamma_{ij}{\left(\hat{\rho }-{\hat{\rho }}^{\left(0\right)}\right)}_{ij}.$$

where $H_{0}$ is the Hamiltonian for this system without the electromagnetic field $\vec{E}\left(t\right)$, $\hat{\rho }$ is the density matrix of single electron state in this system, ${\hat{\rho }}^{\left(0\right)}$ is the unperturbed density matrix, $\Gamma_{ij}$ is the relaxation rate, and $-\hat{M}\vec{E}\left(t\right)=-qz\vec{E}\left(t\right)$ is the perturbation term.

Equation (5) is calculated by the following iterative method:

(6) $$\hat{\rho }\left(t\right)=\sum_{n}{\hat{\rho }}^{\left(n\right)}\left(t\right).$$

with

(7) $$\frac{\partial {\hat{\rho }}_{ij}^{\left(n+1\right)}}{\partial t}=\frac{1}{i\hbar }\left\{{\left[{\hat{H}}_{0},{\hat{\rho }}^{\left(n+1\right)}\right]}_{ij}-i\hbar \Gamma_{ij}{\hat{\rho }}_{ij}^{\left(n+1\right)}\right\}-\frac{1}{i\hbar }{\left[qz,{\hat{\rho }}^{\left(0\right)}\right]}_{ij}\vec{E}\left(t\right).$$

The electric polarization of the system for the first three orders can be expressed as

(8) $$p\left(t\right)=(\varepsilon_{0}\chi^{\left(1\right)}\tilde{E}e^{i\omega t}+\varepsilon_{0}\chi_{0}^{\left(2\right)}|\tilde{E}{|}^{2}+\varepsilon_{0}\chi_{2\omega }^{\left(2\right)}{\tilde{E}}^{2}e^{2i\omega t}+\varepsilon_{0}\chi_{\omega }^{\left(3\right)}{\tilde{E}}^{2}\tilde{E}e^{i\omega t}+\varepsilon_{0}\chi_{3\omega }^{\left(3\right)}{\tilde{E}}^{3}e^{3i\omega t})+c.c.,$$

where $\chi^{\left(1\right)}$, $\chi_{0}^{\left(2\right)}$, $\chi_{2\omega }^{\left(2\right)}$, $\chi_{\omega }^{\left(3\right)}$ and $\chi_{3\omega }^{\left(3\right)}$ are the linear, optical rectification, second harmonic generation susceptibilities and THG susceptibilities, respectively. $\varepsilon_{0}$ is the vacuum dielectric constant. In the present study, we pay attention to the OACs and THG in double semi-V-shaped QWs. By using this approach, the THG susceptibilities and the linear and third-order nonlinear OACs can be obtained analytically as follows:

(9) $$\chi_{3\omega }^{\left(3\right)}=\frac{\sigma_{\nu }}{\varepsilon_{0}}\frac{\mu_{01}\mu_{12}\mu_{23}\mu_{30}}{\left(\hbar \omega -E_{10}-i\hbar \Gamma_{1}\right)\left(2\hbar \omega -E_{20}-i\hbar \Gamma_{1}\right)\left(3\hbar \omega -E_{30}-i\hbar \Gamma_{1}\right)}.$$

where $\sigma_{\nu }$ is the density of electrons in the double semi-V shaped QWs, $\mu_{ij}=|\langle \Psi_{i}\left|ez\right|\Psi_{j}\rangle |$ is the off-diagonal matrix element, $E_{ij}=E_{j}-E_{i}$ is the energy interval of two different electronic states (i,j=0,1,2,3), and ℏω is the incident photon energy. $\Gamma_{k}\left(k=1,2\right)$ is the phenomenological relaxation rate.

In addition, neglecting the higher harmonic terms, the linear and the third-order nonlinear OACs can be obtained by

(10) $$\alpha^{\left(1\right)}\left(\omega \right)=\omega \sqrt{\frac{\varrho }{\varepsilon_{R}}}\frac{|\mu_{10}{|}^{2}\sigma_{\nu }\hbar \Gamma_{2}}{{\left(E_{10}-\hbar \omega \right)}^{2}+{\left(\hbar \Gamma_{2}\right)}^{2}}.$$

(11) $$\begin{array}{lll} \alpha^{\left(3\right)}\left(\omega ,I\right) & = & -\omega \sqrt{\frac{\varrho }{\varepsilon_{R}}}\left(\frac{I}{2\varepsilon_{0}n_{r}c}\right)\frac{|\mu_{10}{|}^{2}\sigma_{\nu }\hbar \Gamma_{2}}{{\left[{\left(E_{10}-\hbar \omega \right)}^{2}+{\left(\hbar \Gamma_{2}\right)}^{2}\right]}^{2}}\left[4\right|\mu_{10}{|}^{2}\\ & - & \frac{|\mu_{11}-\mu_{00}{|}^{2}\left[3E_{10}^{2}-4E_{10}\hbar \omega +\hbar^{2}\left(\omega^{2}-\Gamma_{2}^{2}\right)\right]}{E_{10}^{2}+{\left(\hbar \Gamma_{2}\right)}^{2}}]. \end{array}$$

So, the total absorption coefficient α(ω,I) is given by

(12) $$\alpha \left(\omega ,I\right)=\alpha^{\left(1\right)}\left(\omega \right)+\alpha^{\left(3\right)}\left(\omega \right).$$

where ϱ is the magnetic permeability of the system, $n_{r}$ is the refractive index, $I=2\sqrt{\frac{\varepsilon_{R}}{\varrho }}{\left|E\left(\omega \right)\right|}^{2}=\frac{2n_{r}}{\varrho c}{\left|E\left(\omega \right)\right|}^{2}$ is the incident optical intensity, *c* is the speed of light in free space.

## 3. Results and Discussions

In this section, the linear and nonlinear OACs and THG susceptibilities are calculated numerically for GaAs/Ga_1−*x*_Al_*x*_As double semi-V shaped QWs system. The physical parameters used for the numerical computation are the following: $\sigma_{\nu }=5\times 10^{22}m^{-3}$, $\Gamma_{1}=1/T_{1}$, $T_{1}=0.5$ ps, $\varepsilon_{0}=8.85\times 10^{-12}$ F/m, $n_{r}=3.2$, $\varrho =4\pi \times 10^{-7}$ H/m, $\Gamma_{2}=1/T_{2}$, $T_{2}=0.14$ ps, and I=0.6 MW/cm^2^.

**Figure 2:**
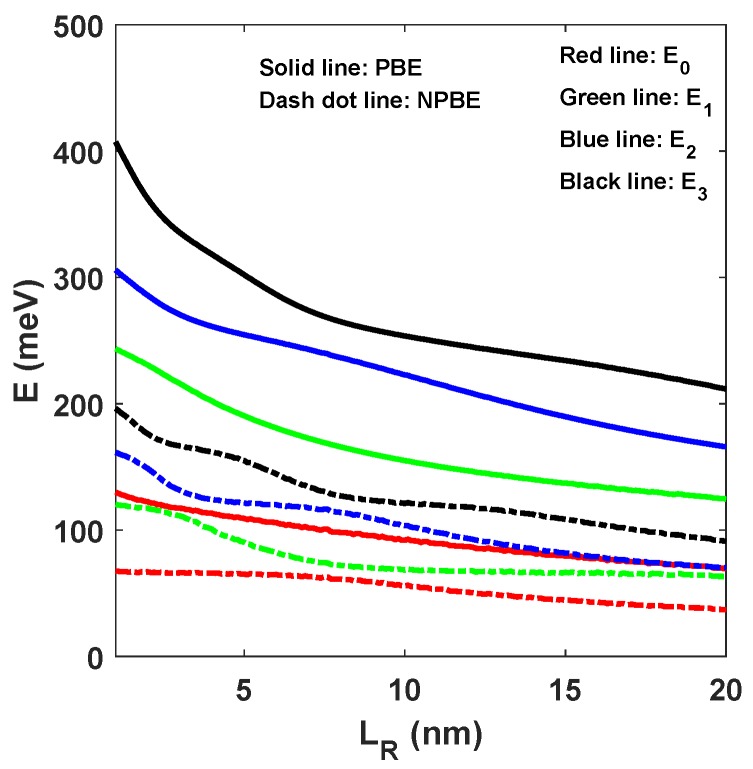
The ground state and the three excited states $E_{1}$, $E_{2}$ and $E_{3}$ as a function of the width of right-hand well $L_{R}$.

The energy levels corresponding to the width of right-hand well $L_{R}$ are depicted in Figure 2. We keep the width of left-hand well $L_{L}=8$ nm and the width of central barrier $L_{B}=1$ nm unchange, and focus on the dependence of these levels on the width of right-hand well $L_{R}$. Also, the influence of NPBE on these levels has been taken into account. As can be seen from the figure, with increase in the width of right-hand well $L_{R}$, the ground and three excited energy levels decay continuously. More important, these energy levels obtained with considering the influence of NPBE are reduced than that obtained without considering the influence of NPBE. Meanwhile, it can be clearly seen that the intervals between adjacent energy levels become closer to each other. So, the NPBE has a significant effect on the quantized energy level positions, which is indispensable for detailed information on the electronic structure, especially in designing resonant tunneling devices.

**Figure 3:**
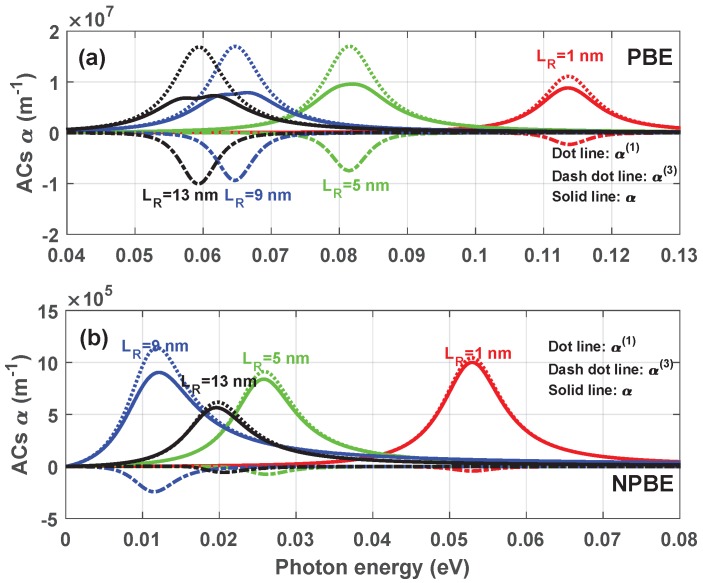
The linear $\alpha^{\left(1\right)}\left(\omega \right)$, the third-order nonlinear $\alpha^{\left(3\right)}\left(\omega \right)$ and the total OACs α(ω) versus the photon energy ℏω with $L_{L}=8$ nm, $L_{B}=1$ nm, for two case: Without (**a**) and with (**b**) considering the influence of NPBE.

Figure 3 illustrates the linear $\alpha^{\left(1\right)}\left(\omega \right)$, the third-order nonlinear $\alpha^{\left(3\right)}\left(\omega \right)$, and the total OACs α(ω) as a function of the photon energy ℏω, with $L_{L}=8$ nm and $L_{B}=1$ nm. Several distinct values of the width of right-hand well $L_{R}$ have been taken into account with and without considering the influence of NPBE, which are illustrated in Figure 3a,b, respectively. From these figures, it can be clearly seen that the $\alpha^{\left(1\right)}\left(\omega \right)$, $\alpha^{\left(3\right)}\left(\omega \right)$ and α(ω) as a function of photon energy ℏω have an prominent peak with a common location, which occurs at $\hbar \omega =E_{10}$ due to the one-photon resonance enhancement. As seen in Figure 3a, As the width of right-hand well $L_{R}$ increases, the resonant peaks shift to the aspect of low energy. This is due to the energy difference $E_{10}$ between the ground state and first excited state decreases when the width of right-hand well $L_{R}$ increases (see Figure 2). Moreover, we can see that the total OACs α(ω) will be significantly split into two peaks with the increase of the width of right-hand well $L_{R}$, which shows the light transparent effect. The wider the width of right-hand well $L_{R}$ is, the more obvious the effect is. That is because when the width of right-hand well $L_{R}$ reaches a certain width, the magnitude of the resonant peaks of the third-order OACs $\alpha^{\left(3\right)}\left(\omega \right)$ increases slightly, and the magnitude of the resonant peaks of the linear OACs $\alpha^{\left(1\right)}\left(\omega \right)$ is almost unchange. Moreover, the total absorption coefficient α(ω) is increased by the linear term, but it is significantly reduced by the third-order nonlinear term. Therefore, the cusp shape in the total OACs α(ω) curves also becomes more prominent with increasing the width of right-hand well $L_{R}$ (see Figure 3a). From Figure 3b, it can be clearly seen that the magnitude of the resonant peaks of the total OACs α(ω) decreases significantly when considering the influence of NPBE. The reason is the correction of NPBE on the wave functions of the electron or the matrix element $\mu_{ij}$ dominated the magnitude of the resonant peaks of OACs (see Equations (10) and (11)). In addition, the resonant peaks suffer an obvious red-shift with the increase of the width of right-hand well when considering the influence of NPBE except for the width of right-hand well $L_{R}=9$ nm. These results originate from the correction of NPBE on the electron energies. It is obvious that the energy levels are close to each other and the energy levels spacing $E_{10}$ is reduced when considering the influence of NPBE(see Figure 2). Furthermore, when considering the influence of NPBE, the saturation will disappear on the total OACs α(ω), and the magnitude of the resonant peaks of the total OACs α(ω) appears a minimum value when the left-hand well and the right-hand well are symmetry.

**Figure 4:**
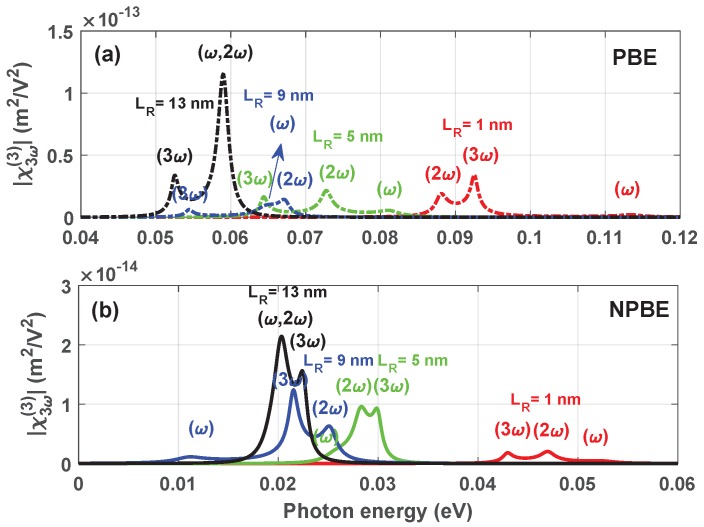
The THG coefficients $|\chi_{3\omega }^{\left(3\right)}|$ as a function of the incident photon frequency ℏω with $L_{L}=8$ nm, $L_{B}=1$ nm, for two case: Without (**a**) and with (**b**) considering the influence of NPBE.

Figure 4 displays the THG coefficients $|\chi_{3\omega }^{\left(3\right)}|$ as a function of the photon energy ℏω without (Figure 4a) and with (Figure 4b) considering the influence of the NPBE for $L_{L}=8$ nm and $L_{B}=1$ nm. One can observe from Figure 4a that (1) There are three resonant peaks corresponding to each one of right-hand well width $L_{R}$, which come from the one photon resonance (ω), two photon resonance (2ω) and three photon resonance (3ω), respectively. (2) When the width of the right-hand well $L_{R}=13$ nm, there are two resonant peaks for $|\chi_{3\omega }^{\left(3\right)}|$, and the magnitude of the THG resonant peaks reaches the maximum. The reason is that some of the differences between these three values $\left(E_{10},E_{20}/2,E_{30}/3\right)$ are more less than the value $\hbar \Gamma_{1}$, which is the damping parameter $\Gamma_{1}$ appearing in the Lorentzian factor of the Equation (9). The superposition between the spectra can enhance the THG $|\chi_{3\omega }^{\left(3\right)}|$, some of the peaks are no longer obvious. (3) It is easily seen that the resonant peaks of the THG $|\chi_{3\omega }^{\left(3\right)}|$ are shift towards the direction of the low energy with increasing the width of the right-hand well $L_{R}$ corresponding to the photon energy $\hbar \omega =E_{10}$, $\hbar \omega =E_{20}/2$ and $\hbar \omega =E_{30}/3$, which is the result of the energy interval $E_{10}$, $E_{20}$ and $E_{30}$ decreasing with increasing the width of the right-hand well $L_{R}$ (see Figure 2). Because each of the spectral width related with THG $|\chi_{3\omega }^{\left(3\right)}|$ is much smaller than the resonance energy interval. Thus the position of resonant peaks can be approximately evaluated by the photon resonance condition. The similar results can be found in the Figure 4b corresponding to the case with considering the influence of the NPBE. But there are some obvious differences as follows: (1) It is worth noting that the magnitude of the THG $|\chi_{3\omega }^{\left(3\right)}|$ resonant peaks is reduced with considering the influence of the NPBE, which shows a significant correction on the magnitude of the THG $|\chi_{3\omega }^{\left(3\right)}|$ resonant peaks. (2) The position of the THG $|\chi_{3\omega }^{\left(3\right)}|$ resonant peaks shift towards lower energies. This trait is attributed to the reduce of the energy interval $E_{10}$, $E_{20}$ and $E_{30}$ compared with the case without considering the influence of the NPBE, which shows the correction on the energy levels of double semi-V shaped QWs system. (3) Compared the case without and with considering the influence of the NPBE, it is to be noted that the position of the one photon resonance (ω) peak, two photon resonance (2ω) peak and three photon resonance (3ω) peak show a complex change due to the energy dependent effective mass m*(E) where the energy value was chosen self-consistently.

**Figure 5:**
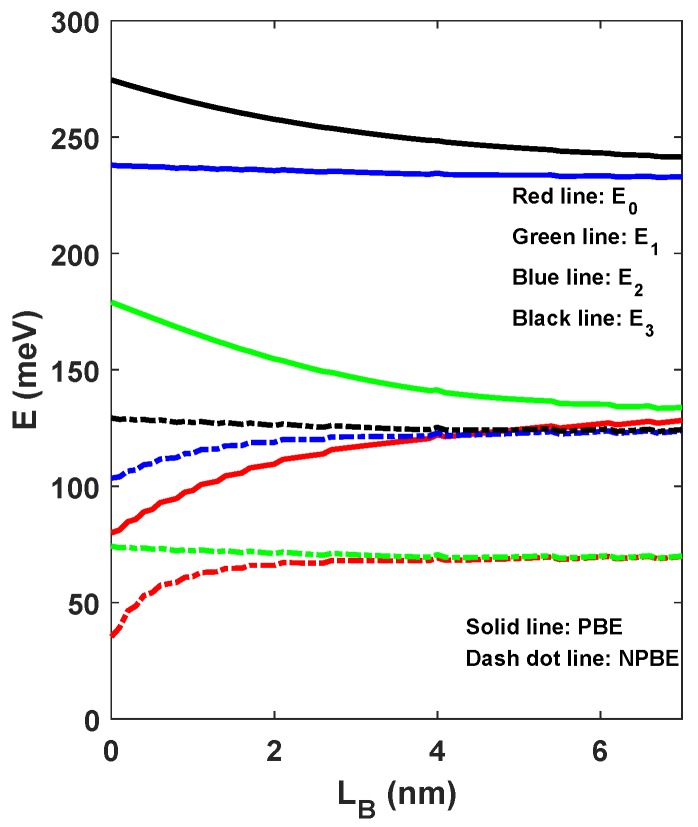
The ground state and the three excited states $E_{1}$, $E_{2}$ and $E_{3}$ as a function of the barrier width $L_{B}$.

The energy levels are depicted in Figure 5 corresponding to the AlGaAs barrier width $L_{B}$, which is varied from 0 nm to 7 nm. We keep the width of left-hand well $L_{L}=8$ nm and the width of left-hand well $L_{R}=8$ nm, and focus on the dependence of these levels on the barrier width $L_{B}$. Also, the influence of NPBE on these levels has been taken into account. When the barrier width $L_{B}=0$, the double semi-V shaped QWs becomes an individual single V shaped QW. The ground state energy level $E_{0}$ slowly increases by increasing barrier width $L_{B}$, which gradually tends to a fixed value when the barrier width $L_{B}>6$. The first excited state energy level $E_{1}$ and the third excited state energy level $E_{3}$ gradually decreases as the barrier width $L_{B}$ increases. The second excited state energy level $E_{2}$ almost keep constant with increasing barrier width $L_{B}$. The energy levels $E_{1}$, $E_{2}$, and $E_{3}$ and the ground state $E_{0}$ have similar changes, which gradually tends to a fixed value when the barrier width $L_{B}>6$. This is because the thick barrier decreases the tunneling between two QWs and makes the coupling QWs behave like an individual single QW with no coupling to the adjacent well [37]. More important, we can observe that these energy levels with considering the influence of the NPBE are significantly reduced compared with the case without considering the influence of the NPBE. Simultaneously, it is obvious that these energy levels have been significantly corrected by the NPBE. The ground state $E_{0}$ and the second excited state $E_{2}$ energy levels slowly increases by increasing barrier width $L_{B}$, which gradually tends to a fixed value when the barrier width $L_{B}>4$. The first excited state $E_{1}$ and the third excited state $E_{3}$ energy levels almost keep constant with increasing barrier width $L_{B}$.

**Figure 6:**
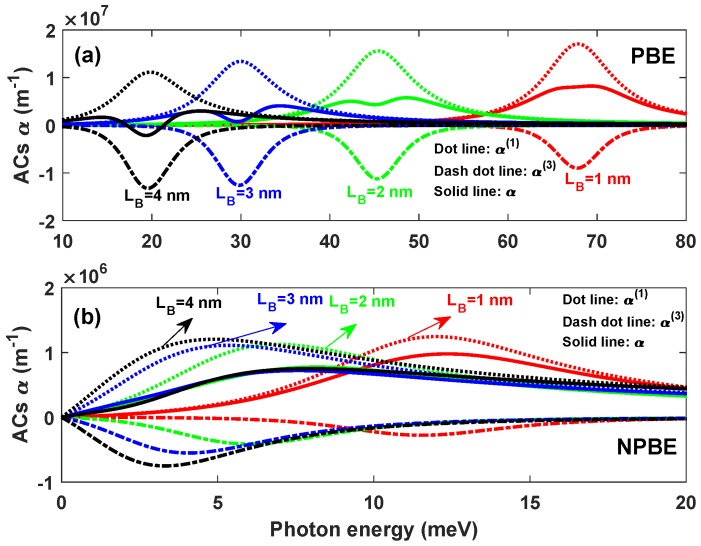
Variations of the linear $\alpha^{\left(1\right)}\left(\omega \right)$, the third-order nonlinear $\alpha^{\left(3\right)}\left(\omega \right)$ and the total OACs α(ω) versus the photon frequency ℏω with $L_{L}=L_{R}=8$ nm, for two case: Without (**a**) and with (**b**) considering the influence of NPBE.

In Figure 6, the linear $\alpha^{\left(1\right)}\left(\omega \right)$, the third-order nonlinear $\alpha^{\left(3\right)}\left(\omega \right)$, and the total OACs α(ω) are plotted as a function of the photon energy ℏω with $L_{L}=L_{R}=8$ nm. Several distinct values of the barrier width $L_{B}$ have been taken into account, for two cases: without (Figure 6a) and with (Figure 6b) considering the influence of the NPBE, respectively. As it can seen from Figure 6a, the resonant peaks of the linear $\alpha^{\left(1\right)}\left(\omega \right)$, the third-order nonlinear $\alpha^{\left(3\right)}\left(\omega \right)$, and the total OACs α(ω) show red shift with increase in barrier width $L_{B}$, which is attributed to the decrease of the energy difference $E_{10}$ with the increase of the barrier width $L_{B}$. All these phenomena agree well with the results discussed above in Figure 5. In addition, the wider the width of barrier width $L_{B}$ is, the more obvious the light transparent effect is. When considering the influence of the NPBE, the magnitude of the resonant peaks of the total OACs α(ω) is significantly reduced, and the saturation of the total OACs α(ω) no longer exists. Additionally the resonant peaks of the linear $\alpha^{\left(1\right)}\left(\omega \right)$, the third-order nonlinear $\alpha^{\left(3\right)}\left(\omega \right)$, and the total OACs α(ω) shift to lower energies. It is due to the decrement in energy difference between the ground state $E_{0}$ and the first excited state $E_{1}$(see Figure 5).

**Figure 7:**
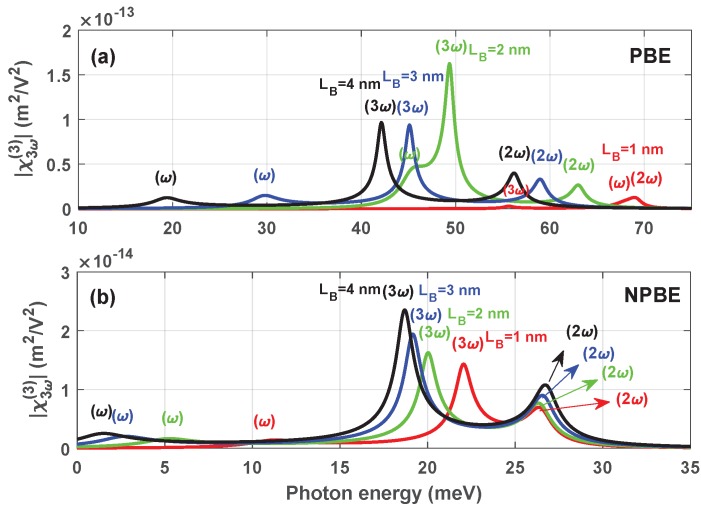
Variations of the THG coefficients $|\chi_{3\omega }^{\left(3\right)}|$ versus the photon frequency ℏω with $L_{L}=L_{R}=8$ nm, for two case: Without (**a**) and with (**b**) considering the influence of NPBE.

In Figure 7, we discuss the THG coefficients $|\chi_{3\omega }^{\left(3\right)}|$ as a function of the photon energy ℏω without (Figure 7a) and with (Figure 7b) considering the influence of the NPBE for $L_{L}=L_{R}=8$ nm. It is seen from the Figure 7a that the resonant peaks will move to the left of the curve when the barrier width $L_{B}$ increases. This phenomenon is attributed to the decreasing of the energy interval $E_{10}$, $E_{20}$ and $E_{30}$ with increasing barrier width $L_{B}$. All these phenomena agree well with above results discussed in Figure 5. The magnitude of the resonant peaks of the THG coefficients $|\chi_{3\omega }^{\left(3\right)}|$ reaches maximum when barrier width $L_{B}=2$ nm. This is because the superposition between the spectra can enhance the THG $|\chi_{3\omega }^{\left(3\right)}|$. In other words, both of the one photon resonance (ω) and the three photon resonance (3ω) happen in the same place $\left(E_{10}=E_{30}/3\right)$. We can find some obvious differences compared with the case with considering the influence of the NPBE (Figure 7b). The resonant peaks of the THG coefficients $|\chi_{3\omega }^{\left(3\right)}|$ appear at lower energies, which is due to the correction of the NPBE, and the magnitude of the resonant peaks of the THG coefficients $|\chi_{3\omega }^{\left(3\right)}|$ is reduced.

## 4. Conclusions

In this paper, the intersub-band OACs and the THG susceptibility with and without considering the influence of the NPBE in GaAs/Ga_1−*x*_Al_*x*_As double semi-V-shaped QWs are theoretically studied. We have used the finite difference method to obtain eigenenergies and eigenfunctions. It is easily observed that the energy levels are significantly reduced when considering the influence of the NPBE. Also the change of energy levels has been corrected by the NPBE. Based on the analysis of these energy levels, meanwhile, we can discuss the intersub-band OACs and the THG susceptibility by using the compact-density-matrix approach. The results show that the intersub-band OACs and the THG susceptibility can reach the magnitude of ∼10^7^ m^−1^ and ∼10^−13^ m^2^/V^2^. However, when considering the influence of the NPBE, the magnitude of the intersub-band OACs and the THG susceptibility is reduced by an order of magnitude. Finally, the resonant peaks and its corresponding to resonant energy are also taken into account. When considering the influence of the NPBE, the position of the resonant peaks of the intersub-band OACs and the THG susceptibility shift to lower energies. Moreover, the position ordering of the THG susceptibility changes with the influence of the NPBE. So the NPBE has a significant effect on the quantized energy level positions and nonlinear optical properties, which is indispensable for detailed information on the electronic structure, especially in designing resonant tunneling devices.
